# Population structure and genetic diversity of *Streptococcus suis* isolates obtained from the United States

**DOI:** 10.3389/fmicb.2023.1250265

**Published:** 2023-09-21

**Authors:** Tracy L. Nicholson, Anwar A. Kalalah, Mark Eppinger

**Affiliations:** ^1^National Animal Disease Center, Agricultural Research Service, United States Department of Agriculture, Ames, IA, United States; ^2^South Texas Center for Emerging Infectious Diseases (STCEID), The University of Texas at San Antonio, San Antonio, TX, United States; ^3^Department of Molecular Microbiology and Immunology (MMI), The University of Texas at San Antonio, San Antonio, TX, United States

**Keywords:** *Streptococcus suis*, whole-genome sequencing (WGS), cgMLST-based typing, comparative genomics, population structure

## Abstract

Diseases caused by the zoonotic pathogen *Streptococcus suis* are an extensive economic problem as well as an animal welfare concern for the global swine industry. Previous studies have evaluated the genomic diversity and population structure of *S. suis* isolates, however, the majority of these studies utilized isolates obtained from countries other than the U.S. This study applied whole genome sequencing and cgMLST-based typing to evaluate the population structure and genetic relatedness among *S. suis* isolates obtained within the U.S. The established high-resolution phylogenomic framework revealed extensive genomic variation and diversity among the sampled *S. suis* isolates, with isolates from the U.S. and from countries outside the U.S. found interspersed in the phylogeny. *S. suis* isolates obtained within the U.S. did not cluster by state or geographic location, however, isolates with similar serotypes, both obtained from within and outside the U.S., generally clustered together. Average nucleotide identity (ANI) values determined for the *S. suis* genomes were extensively broad, approaching the recommended species demarcation value, and correlated with the phylogenetic group distribution of the cgMLST-based tree. Numerous antimicrobial resistance (AMR) elements were identified among both U.S. and non-U.S. isolates with *ble*, *tetO*, and *ermB* genes identified as the most prevalent. The *epf*, *mrp*, and *sly* genes, historically used as markers for virulence potential, were also observed in the genomes of isolates that grouped together forming a subclade of clonal complex 1 (CC1) isolates. Collectively, the data in this report provides critical information needed to address potential biosurveillance needs and insights into the genetic diversity and population structure of *S. suis* isolates obtained within the U.S.

## Introduction

*Streptococcus suis* is one of the leading causes of bacterial infection in swine and contributes to significant economic losses to the swine industry worldwide (Feng et al., 2014; Segura et al., 2014a,b, 2017). *S. suis* causes a spectrum of clinical disease outcomes in pigs including pneumonia, endocarditis, septicemia, and meningitis. *S. suis* is also a zoonotic pathogen capable of causing diseases in humans, mainly arthritis, sepsis, and meningitis (Wertheim et al., 2009a,b; Feng et al., 2014; Segura et al., 2014a,b). Human infections have been either sporadic, and thought to be acquired from penetrating injuries associated with occupational exposure, or epidemic, and associated with consumption of raw or undercooked pork products, primarily in southeast Asia (Gottschalk et al., 2007; Dutkiewicz et al., 2018).

Virulence mechanisms used by *S. suis* are not extensively characterized (Fittipaldi et al., 2012; Segura et al., 2017). Studies addressing *S. suis* virulence have been confounded because different isolates cause a spectrum of disease outcomes ranging from lethal systemic disease to asymptomatic carriage (Segura et al., 2017; Votsch et al., 2018). Virulence-associated factors generally regarded as the most important contributors to disease are the capsular polysaccharide (CPS), muramidase-released protein (*mrp*), extracellular protein factor (*epf*), and suilysin (*sly*) (Fittipaldi et al., 2012; Segura et al., 2014a,b, 2017). However, none of these factors alone correlate with the ability to cause invasive systemic disease (Fittipaldi et al., 2012; Segura et al., 2017). Additionally, invasive clinical isolates obtained from both people and pigs frequently do not harbor all of these factors (Fittipaldi et al., 2012; Feng et al., 2014; Segura et al., 2014a,b, 2017). In addition to being considered a major virulence-factor for *S. suis*, CPS is the basis for the typing system of *S. suis* (Liu et al., 2013). Genotyping isolates using epf + /mrp + /sly + as markers was historically used in epidemiological studies to predict the virulence potential of *S. suis* isolates, particularly serotype 2 isolates obtained from European countries (Gottschalk et al., 1998; Wisselink et al., 2000; Fittipaldi et al., 2012).

Serotyping and multilocus sequence typing (MLST) are the most common methods used to differentiate *S. suis* isolates. Isolates are serotyped based on structural differences in the CPS and to date there are 27 recognized serotypes based on the antigenicity of CPSs and 28 novel CPS loci (Pan et al., 2015; Qiu et al., 2016; Zheng et al., 2017; Huang et al., 2019; Bojarska et al., 2020). Among these, serotype, serotype 2 is regarded as the most virulent and the most frequently isolated serotype from sick pigs (Goyette-Desjardins et al., 2014; Zheng et al., 2014). Additionally, two large outbreaks of human *S. suis* serotype 2 infection have elevated public health concerns (Tang et al., 2006; Yu et al., 2006). A standard MLST scheme has been developed for *S. suis* based on the nucleotide sequences of seven housekeeping genes (King et al., 2002), with isolates subsequently assigned a sequence type (ST) based upon their combination of unique allelic sequences. Worldwide ST1, ST25, and ST28 are the most prevalent STs isolated from swine, the predominance of each ST tends to vary with geographic location (Fittipaldi et al., 2011). For example, in Europe and Asia ST1 strains are more commonly isolated from swine, whereas in North America, ST25 and ST28 are more prevalent (de Greeff et al., 2011; Fittipaldi et al., 2011; Onishi et al., 2012; Schultsz et al., 2012; Zhu et al., 2013; Estrada et al., 2019).

Phylogenetic studies employ whole-genome sequencing (WGS) and data analysis to better understand the epidemiology, origin, and evolution of bacteria (Eppinger et al., 2014; Sahibzada et al., 2017). WGS analysis can be used to assess isolate relatedness or determine genetic characteristics that define subsets of isolates allowing for the determination of phylogenetic relationships among bacterial isolates providing key data for epidemiological studies addressing biosurveillance and risk assessment (Eppinger et al., 2011, 2014; Rusconi et al., 2016; Sahibzada et al., 2017). Numerous studies have undertaken WGS in combination with comparative genomic approaches to evaluate the genomic diversity harbored by *S. suis* isolates (Weinert et al., 2015; Wileman et al., 2019; Hadjirin et al., 2021; Estrada et al., 2022). The U.S. is the third-largest producer and consumer of pork and pork products globally and unfortunately the vast majority of these studies utilize *S. suis* isolates derived from countries other than U.S. (Princivalli et al., 2009; de Greeff et al., 2011; Wang et al., 2011; Goyette-Desjardins et al., 2014; Athey et al., 2015, 2016b; Weinert et al., 2015; Wileman et al., 2019; Scherrer et al., 2020; Guo et al., 2021; Hadjirin et al., 2021; Liang et al., 2021; Estrada et al., 2022). The goal of the current study was to begin to fill this gap by evaluating the genetic relatedness, population structure, and genetic diversity, relating to antimicrobial resistance (AMR) and virulence-associated factors, of *S. suis* isolates obtained within the U.S. using a core genome (cg) MLST-based methodology. Additionally, *S. suis* isolates obtained from various countries outside the U.S. were included in our analyses to provide a broader perspective and examine how the population structure of U.S. *S. suis* isolates compares with isolates obtained from various countries outside the U.S.

## Materials and methods

### Isolate information

*Streptococcus suis* isolates obtained from swine samples collected from pigs throughout the U.S. and submitted to the University of Minnesota Veterinary Diagnostic Laboratory between 2015 and 2017 were selected for the project with an effort to include isolates from as many states and an effort to include as many total isolates as possible without duplication (*n* = 103) (Supplementary Table 1; Nicholson et al., 2020; Nicholson and Shore, 2022). This set was complemented with international *S. suis* isolates for which complete genome assemblies were available in GenBank as of March 22, were selected for the project (*n* = 45) (Supplementary Table 1). All isolates were either obtained from samples collected as part of previous studies or were obtained from samples submitted as part of field case investigations and did not require Institutional Animal Care and Use Committee (IACUC) approval.

### Whole genome sequencing, assembly, and annotation

Draft and complete genomes for *S. suis* isolates obtained from across the United States were generated as previously described (Nicholson et al., 2020; Nicholson and Shore, 2022). Briefly, total genomic DNA was extracted from isolates grown in Trypticase Soy Broth (BD Biosciences, Sparks, MD, USA) using a MasterPure Gram Positive DNA Purification Kit (Lucigen Corporation, Middleton, WI, USA). For draft genome assemblies (*n* = 94), Illumina HiSeq data was obtained from libraries created using the NEB Ultra II DNA Library Prep Kit (New England Biolabs, Ipswich, MA, USA) sequenced on a HiSeq 3000 instrument generating 2 × 150 bp paired-end reads. Sequencing reads were assessed for quality using FastQC.^1^ Reads were randomly subsampled using seqtk^2^ to target a genome coverage of 150x considering an average expected genome length of 2.1 Mbases. Sequence data were assembled using MIRA v. 4.9.6^3^ (Chevreux et al., 1999). The average coverage obtained for each isolate is listed in Supplementary Table 1. To be retained in an assembly, contigs were required to be >1500 bp in length and have a coverage of >66% of the average coverage for the genome.

For closed genome assemblies (*n* = 9), whole genome sequencing was performed using the Pacific Biosciences (PacBio) platform. Library preparation for PacBio sequencing was performed following the PacBio 10-kb insert library preparation protocol. The 10 kb library for each strain was sequenced using the PacBio RSII platform with two SMRT^®^ cells for each isolate. Closed whole-genome assemblies for isolates ISU1606, ISU2660, ISU2414, ISU2515, ISU2614, ISU2714, ISU2812, and SRD478 were generated using the hybrid assembler Unicycler v. 0.4.4 (Wick et al., 2017) software along with both the PacBio sequencing reads and the Illumina MiSeq platform paired-end sequencing reads, which were previously used to obtain draft assemblies for the nine U.S. isolates (Hau et al., 2015). Closed whole-genome assembly for isolate ISU2912 was generated using PacBio SMRT Analysis v 2.3.0,^4^ CANU v. 1.3 (Koren et al., 2017), and GARM v. 0.7.5 (Soto-Jimenez et al., 2014) along with PacBio sequencing reads and the Illumina MiSeq platform paired-end sequencing reads (Hau et al., 2015). Unless specified otherwise, default parameters were used for all software. Final annotations were completed using NCBI’s Prokaryotic Genome Annotation Pipeline (PGAP) v 4.11 (Tatusova et al., 2016). Both draft and complete genomes and sequence reads are available from GenBank and the Sequence Read Archive (SRA). Accession numbers and genome statistics are summarized in Supplementary Table 1.

### MLST and CPS serotype determination

**FIGURE 1:**
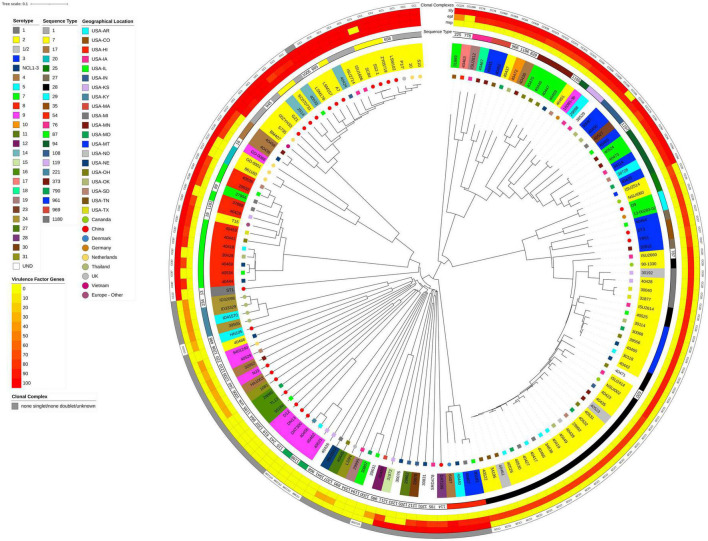
Neighbor-Joining tree based on cgMLST analysis. The closed chromosome of *S. suis* strain ISU1606 (serotype 2; ST 1) served as seed in Ridom SeqSphere + (Junemann et al., 2013). The shared gene inventory was determined at 1,879 genes, comprised of 565 core and 1,314 accessory genes according to the inclusion/exclusion criteria of the SeqSphere + Target Definer. Distance values represent the number of genes with differing allele status in the network. Clonal complex, the nucleotide percentage identity for *epf*, *mrp*, and *sly* converted into a heatmap, ST-classification, serotype, and geographic source is shown from outside in. Serotype for isolates in which the capsule locus was either incomplete or did not match any known serotypes is indicated by UND.

Confirmation of isolates as *S. suis* based on nucleotide sequences of *recN*, determination of MLST, and CPS serotype were performed *in silico* using the automated pipeline developed by Athey et al. (2016a), which uses short-read sequencing data to assign sequence types (STs) based on the typing scheme developed by King et al. (2002) available from the Public Databases for Molecular Typing and Microbial Genome Diversity (Jolley et al., 2018). Amplicon sequencing was subsequently utilized to determine ST for remaining isolates with an unknown and/or uncertain ST. These serotypes were further analyzed *in silico* based on the PCR typing schemes described by Liu et al. (2013) for the classical serotypes, and by Qiu et al. (2016) for the Novel CPS Loci (NCL) serotypes. The CPS locus was either incomplete or did not match any known serotypes for eight isolates (30076, 30831, 38529, 39631, 40426, 40437, 40471, SRD478), and thus designated as undefined (UND) in Figure 1. Using previously reported stringency criteria, a clonal complex (CC) was defined as a group of STs comprised of at least six identical alleles and containing at least three STs [Single Locus Variant (SLV) clustering] (Wang et al., 2015; Scherrer et al., 2020). STs that did not fall into any group were classified as singlets and STs that grouped with only one other ST were classified as doublets. A group of three STs that differ from each other by a single locus but lack other connections were not assigned a CC and classified as having no clear founder.

### MLST schemas and phylogenetic analyses

Assembled genomes were imported into SeqSphere + v. 8.3 (Ridom GmbH, Münster, Germany) for gene-by-gene alignment, allele calling, and comparison (Junemann et al., 2013). A core genome (cg) MLST schema was developed using the closed chromosome of *S. suis* (serotype 2; ST 1) strain ISU1606 (GenBank accession CP030017) as seed. Core genome and accessory MLST targets were identified according to the inclusion/exclusion criteria of the SeqSphere + Target Definer. The allele information from core, accessory, and combined gene sets were used to establish phylogenetic hypotheses using the neighbor-joining method (Saitou and Nei, 1987). The resulting tree was visualized and decorated in iTol v. 6.7.4 (Letunic and Bork, 2021).

### Average nucleotide identity

Average nucleotide identity (ANI) between the designated reference genome *S. suis* strain ISU1606 (serotype 2; ST 1) and the analyzed genomes were calculated using FastANI, based on MinHash mapping (Jain et al., 2018) in Galaxy (Goecks et al., 2010; Afgan et al., 2018).

### Antimicrobial resistance gene profiling

Abricate (Seeman T, Abricate)^5^ was used to identify antimicrobial resistance genes using the AMR gene databases from the Comprehensive Antibiotic Resistance Database (CARD) (Jia et al., 2017), ResFinder [Center for Genomic Epidemiology, (Zankari et al., 2012)] and the NCBI Bacterial Antimicrobial Resistance Reference Gene Database (BioProject Accession PRJNA313047), which was downloaded in April 2019. A minimum percent identity threshold of 80% was used to identify AMR genes in the assembled genomes. Statistical analysis was completed using GraphPad Prism 9.0 (GraphPad Software, Inc., La Jolla, CA, USA). Comparisons of AMR resistance gene frequency between U.S. and non-U.S. isolates were completed using Mann-Whitney tests. A *P*-value less than 0.05 was considered significant.

### Comparative genomic assessment of virulence-associated genes

Genes *mrp* and *sly* were identified by BLASTN searches and the percent identity for each gene was determined for each isolate relative to the *S. suis* strain ISU1606 (serotype 2; ST 1) orthologs. *In silico* PCR using primers from Silva et al. (2006) was used to identify *epf* gene and distinguishment between *epf* or epf*, the long form of extracellular protein factor, based on product size. Percent identity was determined by alignment with either the *epf* gene from strain D282 (accession X71881) or the epf* gene from strain 1890 (accession X71881) (Smith et al., 1993).

## Results

A total of 148 *S. suis* from the U.S. and various countries outside the U.S. were comprehensively analyzed in this study. International isolates were included if complete genome assemblies were publicly available, allowing for the *in silico* screening for AMR- and virulence-factors, as well as sequence type (ST), CPS serotype, and clonal complex (CC) determination. Strain ISU1606 was used as the reference or seed strain because it shares numerous traits and characteristics with *S. suis* strain P1/7 (Holden et al., 2009), which is routinely used a reference strain. ISU1606 and P1/7 are ST1, serotype 2 isolates with similar chromosome size, total number of predicted protein coding sequences (CDSs), G + C content, global synteny, and a similar ability to cause similar clinical disease in swine (Nicholson et al., 2020). To begin evaluating population structure and relatedness among *S. suis* isolates obtained from the U.S. (*n* = 103) and from various countries outside the U.S. (*n* = 45), a neighbor-joining tree was constructed from cgMLST targets using the *S. suis* strain ISU1606 as the seed (Figure 1). The combined cgMLST target gene inventory was determined at 1,879 genes and was comprised of 565 core and 1,314 accessory genes (Supplementary Table 2). The cgMLST-based tree topology shows a high degree of genome plasticity among all the *S. suis* isolates, with the isolates obtained from countries outside the U.S. interspersed throughout the tree (Figure 1). The extensive genome plasticity depicted by the tree topology is reflective of the high genomic variation and diversity previously described for *S. suis* isolates (Princivalli et al., 2009; de Greeff et al., 2011; Weinert et al., 2015; Shelyakin et al., 2019; Guo et al., 2021). Additionally, the *S. suis* isolates obtained within the U.S. did not cluster by state or geographic location (Figure 1).

To examine the nucleotide-level relatedness among the isolates, ANI values were determined for all isolates when compared to *S. suis* strain ISU1606. The ANI values ranged from 99.99% (P1/7) to 94.42% (29995) with an average of 97.12% (Supplementary Table 5). The broad range of ANI values illustrates the extensive genomic variation among the sampled isolates given that ANI values of ≥95% are typically observed for organisms belonging to the same species (Richter and Rossello-Mora, 2009; Kim et al., 2014). The ANI values among the sampled *S. suis* isolates correlate with the phylogenetic group distribution of the cgMLST-based tree (Figure 1). For example, isolates with ANI values ranging 99.99 to 99.70% (P1/7, S10, 10, S735, A7, ZY05719, ISU2714, GZ1, LSM157, 40533, 40424, SC84, SS2-1, SC070731, LSM102, LSM178, JS14, BM407, 05ZYH33) from a distinct cluster with a single branch point (Figure 1 and Table 1). Another example includes the isolates with ANI values ranging 94.70 to 94.42% (ISU2912, 40426, 40445, LS9N, 29995) that are grouped together with only few branch points (Figure 1 and Table 2).

**TABLE 1:** Average nucleotide identity (ANI) values of sampled genomes.

Query genome	ANI value
P1/7	99.99
S10	99.99
10	99.99
S735	99.89
A7	99.88
ZY05719	99.86
ISU2714	99.85
GZ1	99.85
LSM157	99.84
40533	99.84
40424	99.84
SC84	99.82
SS2-1	99.82
SC070731	99.80
LSM102	99.78
LSM178	99.76
JS14	99.73
BM407	99.72
05ZYH33	99.70
ISU2912	94.70
40426	94.62
40445	94.52
LS9N	94.49
29995	94.42

**TABLE 2:** Isolates harboring *epf*, *mrp*, and *sly* genes.

		% Sequence identity					
**Isolate**	**Country of origin**	***epf***	***mrp***	***sly***	**Serotype**	**ST**	**CC**
10	Netherlands	99.92	100	100	2	1	CC1
05ZYH33	China	99.92	100	100	2	7	CC1
A7	China	99.92	100	100	2	7	CC1
BM407	Vietnam	95.65^b^	100	99.93	2	1	CC1
GZ1	China	99.92	100	99.93	2	1	CC1
JS14	China	99.92	77.84^a^	100	14	7	CC1
LSM102	China	99.92	100	100	2	658	CC1
LSM157	China	99.92	99.97^a^	100	2	665	CC1
LSM178	China	99.92	99.97^a^	100	2	1005	None-singlet
P1/7	United Kingdom	99.92	100	100	2	1	CC1
S10	Netherlands	99.92	100	100	2	1	CC1
S735	Netherlands	91.37^b^	99.97	100	2	1	CC1
SC070731	China	99.88	86.2^a^	100	2	7	CC1
SS2-1	China	99.92	100	100	2	7	CC1
ZY05719	China	99.92	100	100	2	7	CC1
40424	United States, IN	99.92	100	100	14	1	CC1
40533	United States, IL	99.92	89.18	100	14	1	CC1
ISU1606	United States, IA	99.90	100	100	2	1	CC1
ISU2714	United States, IA	99.90	100	100	2	1	CC1

^a^Predicted pseudogene based on frameshift. ^b^epf* encoding the long form of extracellular protein factor (EF*).

Two additional cgMLST-based trees were constructed based on the core genes (Supplementary Figure 1) and the accessory genes only (Supplementary Figure 2). The resulting core-genome based tree resulted in an overall tree topology similar to the cgMLST-based tree (Figure 1 and Supplementary Figure 1). This finding is not surprising as the phylogenetic input is dominated by the shared core genes rather than genes classified as accessory. The accessory-based tree showed in general similar relatedness of isolates, however, reposition of some isolates were observed in the same branches (Figure 1 and Supplementary Figure 2). For example, isolates ZY05719, SS2-1, and SC84 are clustered together from a branch point in the cgMLST-based tree (Figure 1) and in the accessory-based tree these isolates are also clustered together from a branch point but in a repositioned order (Supplementary Figure 2).

Overall isolates with similar CCs and serotypes, obtained from within and outside the U.S., were observed were observed to cluster together (Figure 1). For example, a group of 13 isolates mainly consisting of CC87 (ST19, ST87, and ST1181) were clustered together in Figure 1. Eleven of these 13 isolates were CPS serotype 8. The two non-serotype 8 isolates were T15 (serotype 2, ST19, and CC87) and 37904 (serotype 7, ST89, and CC87) (Figure 1). Additionally, serotype 2 isolates mainly clustered together and are partitioned in two distinct groups. One group largely consists of CC28 (ST28, ST620, ST787, ST961, and ST1180) isolates and a second group that mainly consisting of CC1 (ST1, ST7, ST658, ST665) isolates (Figure 1). The CC28- dominated group forms a large clade of 45 isolates, all serotype 2, except for nine (Figure 1). Of the nine non-serotype 2 isolates, D9 and 13-00285-02 (serotype 7, ST29, and CC29) cluster together and next to four serotype 3 and CC27 isolates: 40464, ST3, YB51, and 30815 (Figure 1). These two small clusters separate isolates ISU2514 and NSU1060 both serotype 2, ST25, and CC25 from the other thirty-one isolates, which were serotype 2 and CC28. Twenty-one of these serotype 2 isolates are ST28 and five are ST961. Four isolates within this subclade of CC28 isolates are non-serotype 2 isolates. These non-serotype 2 isolates are 30192, 40523, and 40461, all serotype 1/2, and isolate 40471, which harbor an undetermined serotype (Figure 1).

The other group of serotype 2 isolates consisted of twenty-five isolates, which is further divided into two subclades. The smaller subclade consists of five CC17 (ST17 and ST20) and CC16 (ST16) isolates and included isolates 861160 and GD-0001 (both serotype 2, ST20, CC17), isolate GD-0088 (serotype 9, ST16, CC16), as well as isolates 40439 and 40458 (both serotype 4, ST17, CC17). The larger subclade consists of 20 isolates, all CC1 (ST1, ST7, ST658, ST665) except for isolate LSM178, an ST1005 isolate not assigned to a CC and therefore listed as a singlet. Four of the twenty isolates are U.S. isolates (40424, 40533, ISU1606, and ISU2714), while the majority (16/20) of isolates within this subclade are non-U.S. isolates. Additionally, 17 isolates of this subclade were serotype 2 and three isolates were serotype 14 (40424, 40533, and JS14). Isolates within this subclade were mainly ST1 (9/20) or ST7 (6/20).

**FIGURE 2:**
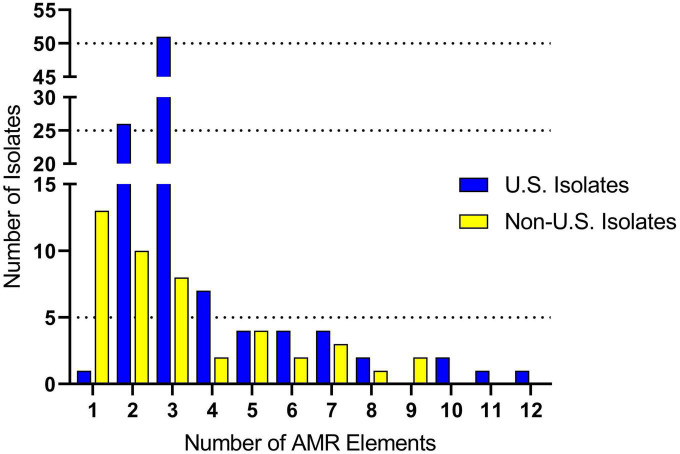
Antimicrobial resistance (AMR) gene frequency among *S. suis* isolates. The *x*-axis indicates the number of AMR genes harbored by a single isolate. The *y*-axis indicates the number of isolates identified harboring each the respective number of AMR genes.

Genomes were screened for chromosomal mutations and genes conferring antimicrobial resistance. For the U.S. isolates, the number of AMR elements harbored by an individual isolate ranged from one to twelve (Figure 2 and Supplementary Table 3). The average number of AMR elements harbored by an individual U.S. isolate was three (Figure 2 and Supplementary Table 3). The number of AMR elements harbored by an individual isolate ranged from one to nine for the non-U.S. isolates (Figure 2 and Supplementary Table 3). The average number of AMR elements harbored by an individual non-U.S. isolate was two, which was significantly (*P* = 0.038) lower than number of AMR elements harbored by an individual U.S. isolate (Figure 2 and Supplementary Table 3). While numerous AMR elements were identified, the most prevalent were *ble* (100% in both U.S. and non-U.S.) followed by *tetO* (87% U.S. and 49% non-U.S.), and *ermB* (65%, U.S. and 49% non-U.S.). Both the *tetO* and *ermB loci* are absent in 27 genomes, eleven of which were serotype 2 isolates grouped within the larger subclade CC1 isolates in Figure 1.

Genes *epf*, *mrp*, and *sly* have historically been used to predict the virulence potential of *S. suis* (Gottschalk et al., 1998; Wisselink et al., 2000; Fittipaldi et al., 2012). Thus, all genomes were screened for the presence of these loci and the percent identity relative to the reference orthologs was determined. The *epf* gene is present in four U.S. isolates (40424,40533, ISU1606, and ISU2714) with a 99.9% identity to the reference ortholog. None of the four isolates encode the long form of extracellular protein factor (EF*), which can be expressed by some serotype 2 isolates and produce a high molecular weight variant (>110 kDa) (Galina et al., 1996; Gottschalk et al., 1998). This gene is further found in sixteen non-U.S. isolates with a sequence identity range from 91.37 to 99.9%. Two of these non-U.S. isolates, BM407 and S735, carry epf* (Galina et al., 1996; Gottschalk et al., 1998). The *mrp* gene was found in 86 U.S. isolates and the nucleotide sequence diversity ranged from 31.47 to 100%, with an average sequence identity of 80.21% (Supplementary Table 4). Among non-U.S. isolates, the *mrp* gene was found in 37 isolates and, similar to U.S. isolates, a high nucleotide sequence diversity was observed ranging from 29.76 to 100% with an average 73.72% sequence identity (Supplementary Table 4). The *mrp* gene for nine (13-00283-02, D9, JS14, LSM157, LSM178, NSUI060, SC070731, ST3, T15) of the 37 isolates was predicted to encode a frameshift resulting in a pseudogene. Extensive nucleotide diversity with the *mrp* gene has been previously reported (Vecht et al., 1991; Galina et al., 1996; Baums and Valentin-Weigand, 2009). The *sly* gene was found in 45 U.S. isolates and is highly conserved, with a sequence identity range from 99.2–100% and an average 99.78% sequence identity (Supplementary Table 4). Twenty-four non-U.S. isolates carry the *sly* gene and, similar to U.S. isolates, feature high nucleotide sequence diversity with an average 99.9% sequence identity (99.4–100%) (Supplementary Table 4).

Among the total 148 analyzed isolates, 19 [15 non-U.S. and four U.S. isolates (Table 2) carry all three *epf*, *mrp*, and *sly* genes]. These nineteen isolates form a larger subclade mainly comprised of serotype 2 isolates, as previously mentioned (Figure 1). As stated above, all isolates are CC1 except for isolate LSM178, an ST1005 isolate not assigned to a CC and therefore listed as a singlet (Table 2). Sixteen of these isolates were serotype 2 and three isolates were serotype 14 (40424, 40533, and JS14) and the majority of these isolates were ST1 (10/19) or ST7 (6/19) (Figure 1 and Table 2).

## Discussion

Diseases caused by *S. suis* are a substantial economic problem as well as an animal welfare concern for the global swine industry. Additionally, there are increasing public health concerns regarding the zoonotic potential of *S. suis.* While numerous studies have employed a combination of comparative genomic approaches to assess the genomic diversity and population structure of *S. suis* isolates globally, the overwhelming majority of these studies utilize *S. suis* isolates obtained from countries other than the U.S. (Princivalli et al., 2009; de Greeff et al., 2011; Wang et al., 2011; Goyette-Desjardins et al., 2014; Athey et al., 2015, 2016b; Weinert et al., 2015; Wileman et al., 2019; Scherrer et al., 2020; Guo et al., 2021; Hadjirin et al., 2021; Liang et al., 2021; Estrada et al., 2022). In this report we examined the genomic diversity, phylogenetic relatedness, and population structure of *S. suis* isolates obtained within the U.S. by utilizing a cgMLST approach. Additionally, *S. suis* isolates obtained from various countries outside the U.S. were included in our analyses to provide a broader perspective and examine how the population structure of U.S. *S. suis* isolates compares with isolates obtained from countries outside the U.S.

The resulting phylogenetic tree from the cgMLST-based typing method demonstrated the extensive genomic variation and diversity among all the sampled *S. suis* isolates, with isolates obtained from countries outside the U.S. found interspersed throughout the cgMLST-based tree. The observed extensive genome plasticity in the established cgMLST-phylogeny is reflective of the high genomic variation and diversity previously described for *S. suis* (Princivalli et al., 2009; de Greeff et al., 2011; Weinert et al., 2015; Shelyakin et al., 2019; Guo et al., 2021). In fact, *S. suis* is considered to have an incompletely defined or open pan-genome since the number of unique or accessory genes has increased and the number of conserved genes have decreased as more genome sequences become available (Wang et al., 2011; Shelyakin et al., 2019; Guo et al., 2021).

Average nucleotide identity has increasingly been utilized as a robust method for assessing genomic similarity between two genomes, with organisms belonging to the same species typically having ANI values of ≥95%. The ANI values determined for the *S. suis* isolates in this study were extensively broad, ranging from 99.99% (P1/7) to 94.42% (29995), and approach the recommended species demarcation value (Richter and Rossello-Mora, 2009; Kim et al., 2014). The broad range of ANI values observed illustrate the extensive genomic variation among the isolates and correlates with the group distribution in the cgMLST-based tree. The plasticity in genome composition for the sampled *S. suis* isolates is also evidenced in the genome size, ranging from ISU2912 with a size of 2,720,381 bp and thus 31.17% larger than ISU1606 to S735 with a size of 1,980,887 bp (4.49% smaller than ISU1606).

While the *S. suis* isolates obtained within the U.S. did not cluster by state or geographic location, in general all isolates with similar serotypes, regardless of their origins, cluster together in the cgMLST phylogeny. An exception to this general trend were the serotype 2 isolates, which clustered into two distinct groups. One group mostly consisted of ST28 and ST961 and contained three serotype 1/2 isolates and one isolate with an undetermined serotype. The second group, consisting of ST1 and ST7, had three serotype 14 isolates clustered within the group. The clustering of these isolates harboring the 1/2, 2, and 14 serotypes is reasonable and consistent with the high degree of similarly among these serotypes. Specifically, the CPS loci of serotypes 1 and 14 and serotypes 2 and 1/2 are almost identical and most current PCR methods are unable to differentiate these specific serotypes (Okura et al., 2013). Their CPS structure differs by the substitution of only a galactose (Gal) for a N-acetylgalactosamine (GalNAc) due to a single nucleotide polymorphism in the glycosyltransferase *cpsK* gene (Van Calsteren et al., 2016; Roy et al., 2017). Further, serotype switching of field strains of serotypes 2 and 1/2, and 14 and 1, solely by replacing the amino acid 161 of CpsK has been demonstrated (Roy et al., 2017). In contrast, the CPS loci of all other serotypes are greatly different (Pan et al., 2015; Zheng et al., 2017; Huang et al., 2019).

The clustering of serotype 2 isolates into two distinct groups is supported by previous work that inferred the population structure using MLST and CC approaches (Okura et al., 2013; Hatrongjit et al., 2020; Liang et al., 2021). For example, one branch of serotype 2 isolates within the cgMLST-based tree consisting of ST25, ST27, ST35 isolates further branched into ST28, ST620, ST961, and ST1180, which are the STs comprising one of the serotype 2 groups. Based on CC methods, CC25 (consisting of ST25 isolates), CC27 (consisting of ST27 and ST35 isolates), and CC28 (consisting of ST28, ST620, ST961, ST1180, and ST787) are all linked together (Okura et al., 2013; Hatrongjit et al., 2020; Liang et al., 2021). Additionally, CC1 (consisting of ST1 isolates) and CC7 (consisting of ST7 isolates) are linked (Okura et al., 2013; Hatrongjit et al., 2020; Liang et al., 2021). Consistent with this, the second branch of serotype 2 isolates is comprised of ST1 and ST7 isolates. The results demonstrate that overall, there is a high degree of similarity and congruence between *S. suis* population structure based on previously CC methods and the cgMLST-based strategy utilized in this study. However, the established cgMLST phylogenies provide additional resolution as evident by the observed expanded branching and network.

In addition to the phylogenetic relatedness and population structure of *S. suis* isolates, AMR elements and virulence-associated genes were analyzed. Numerous AMR elements were identified among both U.S. and non-U.S, the most prevalent were *ble*, *tetO*, and *ermB*. However, the *tetO* and *ermB* genes were not observed in the genomes of isolates grouped within the larger subclade CC1 isolates shown in Figure 1. The *epf*, *mrp*, and *sly* genes, historically used as markers for virulence potential, were also observed in the genomes of isolates grouped within the larger subclade CC1 isolates shown in Figure 1.

Taken together, the data provided in this report demonstrate that the extensive genomic variation and diversity observed among *S. suis* isolates globally is also reflected among *S. suis* isolates obtained within the U.S. A high-resolution phylogenetic framework for *S. suis* can help provide data needed to improve risk assessments and address public health concerns associated with occupational exposure to *S. suis*.

## Data availability statement

The datasets presented in this study can be found in online repositories. The names of the repository/repositories and accession number(s) can be found in the article/Supplementary material.

## Ethics statement

Ethical approval was not required for the study involving animals in accordance with the local legislation and institutional requirements because all isolates were either obtained from samples collected as part of previous studies or were obtained from samples submitted as part of field case investigations and did not require Institutional Animal Care and Use Committee (IACUC) approval.

## Author contributions

All authors conceived and designed the experiments, performed the experiments, analyzed the data, contributed reagents, materials, and analysis tools, wrote the manuscript, approval of the final version to be published, and agreed to be accountable for all aspects of the work.

## Footnotes

1
https://www.bioinformatics.babraham.ac.uk/projects/fastqc/

2
https://github.com/lh3/seqtk

3
https://mira-assembler.sourceforge.net/docs/

4
https://www.pacb.com/support/software-downloads/

5 Github http://github.com/tseemann/abricate.

## Supplementary material

Supplementary Figure 1 Core gene MLST. The closed chromosome of *S. suis* strain ISU1606 (serotype 2; ST 1) served as seed for all in Ridom SeqSphere + (Junemann et al., 2013). The core gene inventory was determined at 565 genes according to the inclusion/exclusion criteria of the SeqSphere + Target Definer. Distance values represent the number of genes with differing allele status in the network. ST-classification, serotype, and geographic source is shown from outside in. Serotype for isolates in which the capsule locus was either incomplete or did not match any known serotypes is indicated by UND.

Supplementary Figure 2 Accessory gene MLST. The closed chromosome of *S. suis* strain ISU1606 (serotype 2; ST 1) served as seed in Ridom SeqSphere + (Junemann et al., 2013). The accessory gene inventory was determined at 1,314 genes according to the inclusion/exclusion criteria of the SeqSphere + Target Definer. Distance values represent the number of genes with differing allele status in the network. ST-classification, serotype, and geographic source is shown from outside in. Serotype for isolates in which the capsule locus was either incomplete or did not match any known serotypes is indicated by UND.

Supplementary Table 1 Isolate information. The table list the *S. suis* isolates obtained from across the U.S. (sheet 1) and from various countries outside the U.S. (sheet 2), along with serotype, ST, accession numbers, and genome statistics. Serotype for isolates in which the capsule locus was either incomplete or did not match any known serotypes is indicated by UND.

Supplementary Table 2 cgMLST allelic changes. The table lists the total of 253,599 allelic changes identified in the 1,879 cgMLST gene inventory, identified by their locus tag numbers. Allelic change positions and annotations correspond to reference *S. suis* strain ISU1606.

Supplementary Table 3 Antimicrobial resistance (AMR) gene distribution. The table lists AMR gene elements identified in all *S. suis* isolates obtained from across the U.S. (sheet 1) and from various countries outside the U.S. (sheet 2).

Supplementary Table 4 Virulence-associated gene distribution. The table lists virulence-associated genes (*epf*, *mrp*, and *sly*) identified in all *S. suis* isolates obtained from across the U.S. (sheet 1) and from various countries outside the U.S. (sheet 2).

Supplementary Table 5 Average nucleotide identity (ANI) values of cgMLST sampled genomes. The table lists ANI values between the designated reference genome *S. suis* strain ISU1606 and all queried genomes.
