# Supplementary figures and images for: Population structure and genetic diversity of Streptococcus suis isolates obtained from the United States
Source: Front Microbiol. 2023 Sep 21;14:1250265. doi: 10.3389/fmicb.2023.1250265 (PMC10551183; doi:10.3389/fmicb.2023.1250265)

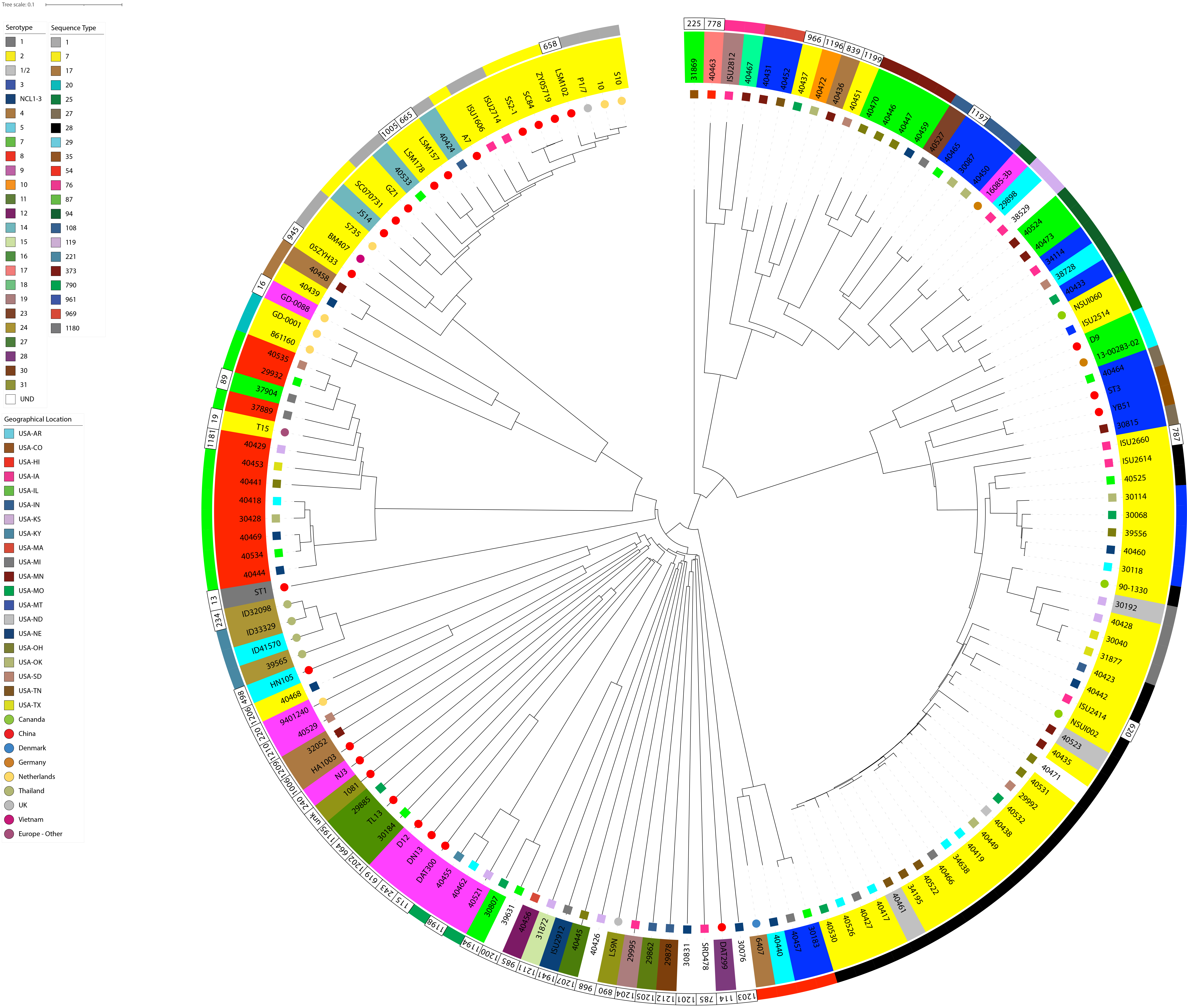

Supplement: Supplementary Figure 2 — Accessory gene MLST. The closed chromosome of S. suis strain ISU1606 (serotype 2; ST 1) served as seed in Ridom SeqSphere + (Junemann et al., 2013). The accessory gene inventory was determined at 1,314 genes according to the inclusion/exclusion criteria of the SeqSphere + Target Definer. Distance values represent the number of genes with differing allele status in the network. ST-classification, serotype, and geographic source is shown from outside in. Serotype for isolates in which the capsule locus was either incomplete or did not match any known serotypes is indicated by UND. [file Image_2.JPEG]
